# A Loose Domain Swapping Organization Confers a Remarkable Stability to the Dimeric Structure of the Arginine Binding Protein from *Thermotoga maritima*

**DOI:** 10.1371/journal.pone.0096560

**Published:** 2014-05-15

**Authors:** Alessia Ruggiero, Jonathan D. Dattelbaum, Maria Staiano, Rita Berisio, Sabato D'Auria, Luigi Vitagliano

**Affiliations:** 1 Institute of Biostructures and Bioimaging, CNR, Napoli, Italy; 2 Department of Chemistry, University of Richmond, Richmond, Virginia, United States of America; 3 Laboratory for Molecular Sensing, IBP-CNR, Naples, Italy; University of South Florida College of Medicine, United States of America

## Abstract

The arginine binding protein from *Thermatoga maritima* (TmArgBP), a substrate binding protein (SBP) involved in the ABC system of solute transport, presents a number of remarkable properties. These include an extraordinary stability to temperature and chemical denaturants and the tendency to form multimeric structures, an uncommon feature among SBPs involved in solute transport. Here we report a biophysical and structural characterization of the TmArgBP dimer. Our data indicate that the dimer of the protein is endowed with a remarkable stability since its full dissociation requires high temperature as well as SDS and urea at high concentrations. In order to elucidate the atomic level structural properties of this intriguing protein, we determined the crystallographic structures of the apo and the arginine-bound forms of TmArgBP using MAD and SAD methods, respectively. The comparison of the liganded and unliganded models demonstrates that TmArgBP tertiary structure undergoes a very large structural re-organization upon arginine binding. This transition follows the Venus Fly-trap mechanism, although the entity of the re-organization observed in TmArgBP is larger than that observed in homologous proteins. Intriguingly, TmArgBP dimerizes through the swapping of the C-terminal helix. This dimer is stabilized exclusively by the interactions established by the swapping helix. Therefore, the TmArgBP dimer combines a high level of stability and conformational freedom. The structure of the TmArgBP dimer represents an uncommon example of large tertiary structure variations amplified at quaternary structure level by domain swapping. Although the biological relevance of the dimer needs further assessments, molecular modelling suggests that the two TmArgBP subunits may simultaneously interact with two distinct ABC transporters. Moreover, the present protein structures provide some clues about the determinants of the extraordinary stability of the biomolecule. The availability of an accurate 3D model represents a powerful tool for the design of new TmArgBP suited for biotechnological applications.

## Introduction

Structural plasticity is a fundamental feature of proteins. Despite the paradigm that associates protein sequences to well-defined 3-dimensional structures [1], it is commonly accepted that proteins are often endowed with repertoires of distinct structural states. Protein structural transitions play major roles in several biological processes, including protein-protein recognition, protein-ligand binding, and signalling.

The mechanism of small molecule transport across biological membranes represents one of the most striking examples that highlights the role of protein flexibility in cellular processes. This transport is generally carried out by intricate systems in which dynamical events are essential for the transfer of information from one protein component to the other. Prototypical examples in this context are represented by the ATP-binding cassette (ABC) systems that play a fundamental role in the import of essential nutrients and in the export of toxic molecules in bacteria [2]. Canonical ABC cassette systems share a common structural organization comprised of two transmembrane domains (TMDs) that form the translocation pore and two nucleotide-binding domains (NBDs) that hydrolyze ATP. The action of these systems generally depends on the presence of extra-cytoplasmic ancillary proteins, denoted as substrate binding proteins (SBPs), which recognize substrates with high affinity and deliver them to the TMD domains [2]. Interestingly, the structural characterization of the proteins involved in this process has revealed that these intricate molecular machines use complex dynamic mechanisms to fulfil their functions [3], [4]. Indeed, SBPs undergo large structural rearrangement upon substrate binding according to the so-called Venus fly-trap mechanism [5]. The interaction of the substrate-bound form of SBPs with the periplasmic peptide regions of the TMDs of the cognate ABC transporter, initiates the transport process. Although the atomic details of this cascade of events are not fully understood, it is commonly accepted that the entire process relies on large conformational transitions of both TMD and NBD moieties of the ABC systems.

The ability of SBPs associated with the ABC transport machinery to bind a variety of different ligands and their intrinsic structural versatility have made them very attractive systems for the development of platforms based on fluorescent protein biosensors for many naturally-occurring ligands. Indeed, by *ad hoc* re-engineering of their binding pockets it is possible to generate proteins able to recognize specific analytes for which sensors are eagerly needed.

As ideal biosensors are expected to have remarkable stability, we turned our attention to proteins isolated from thermophilic organisms. We identified the arginine binding protein form *Thermatoga maritima* (TmArgBP) as an ideal system for arginine detection [6], [7], [8], [9], [10]. Arginine sensing is extremely important since argininemia is a debilitating inherited condition, characterized by a gradual accumulation of arginine and ammonia in the blood, whose diagnosis is crucial for effective medical intervention [11]. The biochemical and biophysical characterization of the protein has shown that TmArgBP presents a number of remarkable properties. These include an extraordinary stability to both temperature and chemical denaturants [9]. Additionally, in contrast to the vast majority of SBP that operate as monomers, TmArgBP forms multimeric assemblies at room temperature [7], [9].

In order to elucidate the atomic level structural properties of this intriguing protein, we determined the crystallographic structures of both the apo and the arginine-bound forms of the protein. The analysis of these structures reveals some unexpected features and provides a solid structural framework for interpreting the biochemical and biophysical properties of the protein. The availability of accurate three-dimensional models for the different states of the protein strongly facilitates the design of variants that can act as durable and highly specific sensors for arginine.

## Results

### Detection and stability of TmArgBP dimer in solution

**Figure 1:**
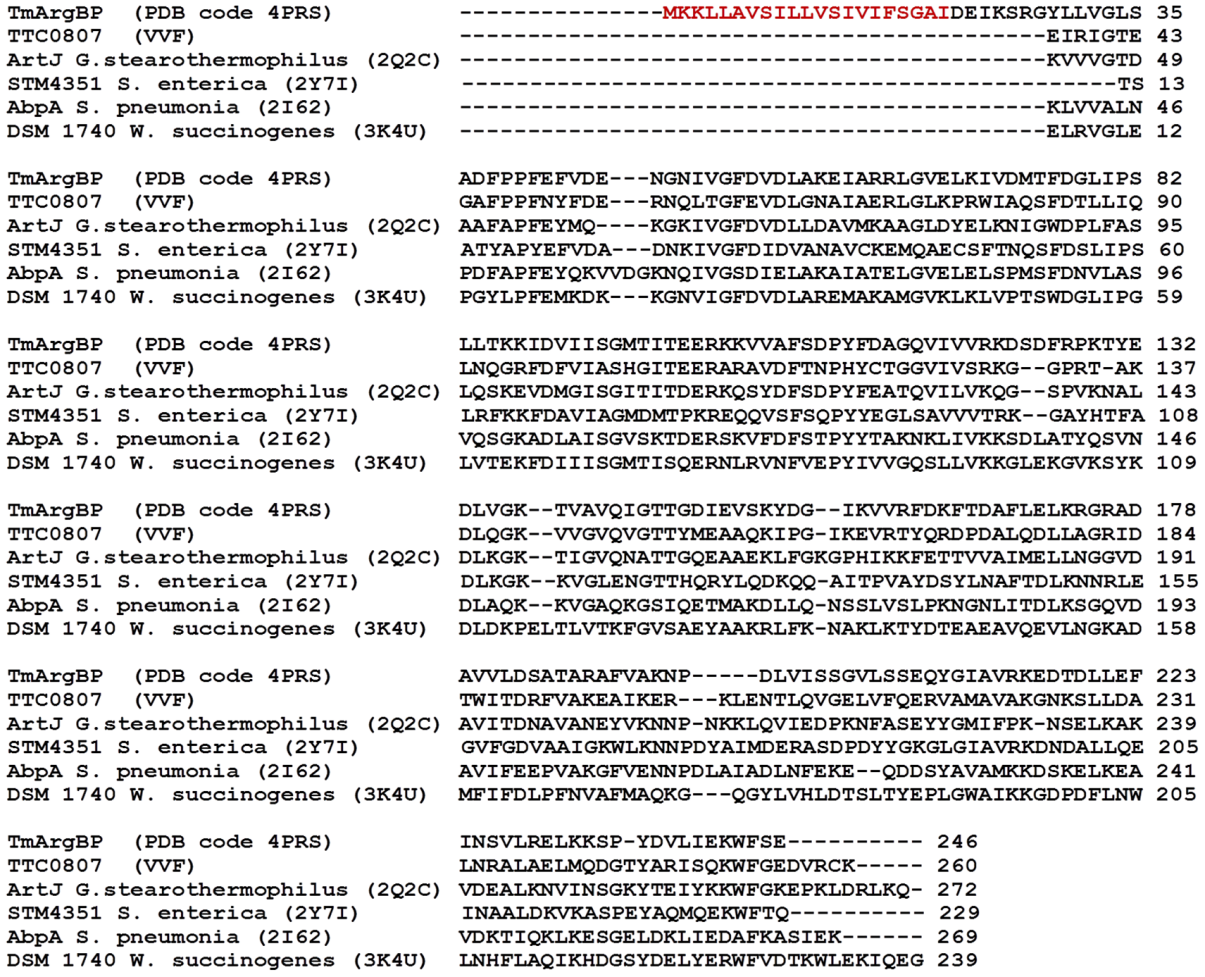
Multiple sequence alignment of TmArgBP with known homologues. For each protein the PDB code is reported in parenthesis. The signal peptide of TmArgBP is drawn in red. For the other proteins only the binding domain is reported.

TmArgBP is comprised of 246 amino acid residues, including a periplasmic signal localization peptide at its N-terminus. The analysis of the protein sequence performed using the TMHMM server (http://www.cbs.dtu.dk/services/TMHMM/) clearly indicates that residues 4 to 21 form a trans-membrane helix (Figure S5 in File S1). The first three residues are expected to be outside the membrane whereas the periplasmatic portion corresponds to the residues 22–246 (Figure 1). We expressed and purified the full-length protein deprived of the membrane localization signal (residues 20–246), in a soluble and homogeneous form that was amenable for both solution and crystallography studies.

**Figure 2:**
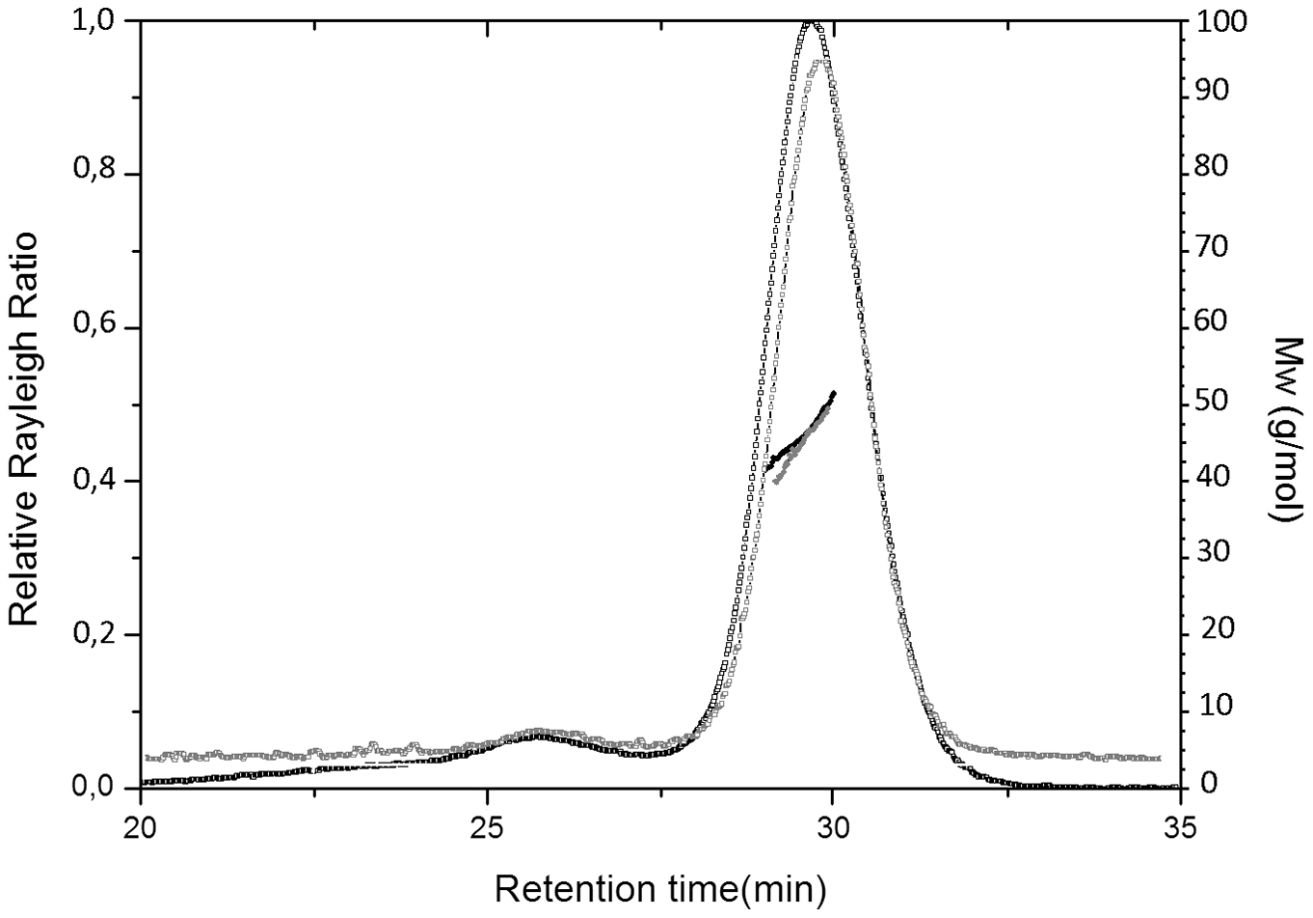
Analytical SEC-MALS of TmArgBP. The black and grey curves represent the Rayleigh ratio (left scale) of HoloTmArgBP and ApoTmArgBP, respectively; both are plotted against the retention time. Molecular masses are reported with the same colour code. In both experiments, average molecular masses values correspond to a dimeric state of the protein.

The oligomerization state of the protein was initially characterized by performing gel filtration and light scattering measurements (Figure 2). In line with previous reports [7], [9], gel filtration analysis indicates that the protein adopts different aggregation states (data not shown). Although aggregates with higher molecular masses were detected, we focused our attention on the main component. The analysis of this component by light scattering provided a weight-average molar mass of 50.3±0.2 and 49.6±0.1 kDa, for HoloTmArgBP and ApoTmArgBP, respectively. Taking into account the theoretical mass of the TmArgBP monomer (25267 Da), these data clearly indicate a dimeric organization of both the apo and the holo forms in solution (Figure 2). We also observed that this dimer does not undergo dissociation or further aggregation with time.

**Figure 3:**
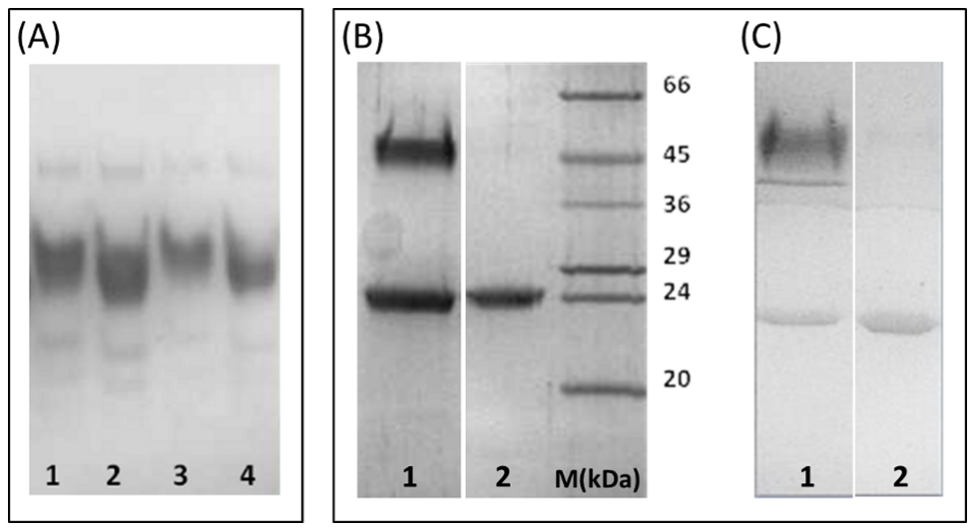
Stability of the TmArgBP dimer. (A) Native PAGE electrophoresis of HoloTmArgBP and ApoTmArgBP. Lanes 1 and 2 contain HoloTmArgBP and ApoTmArgBP, respectively. The same experiments were carried out (Lanes 3 and 4) in the presence of 4M urea. (B) SDS PAGE upon treatment of Holo-TmArgBP and (C) Apo-TmArgBP with increasing urea concentrations. Lanes 1 and 2 contain urea concentrations 0 and 8 M, respectively. The same markers were used in the two experiments.

Native gel electrophoresis experiments confirms the presence of a single predominant species, likely the dimer, which is stable in the presence of 4 M urea (Figure 3A). In order to gain further insights into the stability of the TmArgBP dimer, the protein was heated in the presence denaturants (Figure 3). In particular, SDS-PAGE (15%) was performed after heating the protein at 100°C for 5 minutes and indicates the coexistence of the dimeric/monomeric forms for both ApoTmArgBP and HoloTmArgBP. Interestingly, the dimer is fully dissociated only with the addition of urea at high concentrations (Figure 3B, C). However, the dissociation of the dimer is reversible, since this form can be restored upon urea dilution (data not shown). These results demonstrate that the dimeric organization of TmArgBP is endowed with a remarkable stability, which is independent of the binding state of the protein. The purified dimeric form of TmArgBP was used in the subsequent structural characterization.

### The overall structure of ApoTmArgBP

All attempts to solve the structure of the apo/holo forms of TmArgBP using molecular replacement were unsuccessful. This was likely due to the large unit cell of the holo form and to the high flexibility of the apo form. Therefore, a seleno-methionine derivative of TmArgBP was prepared to set up anomalous dispersion experiments. The structure of the apo form was solved by performing multi-wavelength anomalous diffraction experiments, collecting data at three distinct wavelengths (Table 1).

**Table 1:** Data collection statistics.

	SeMet derivative Holo	SeMet derivative Apo		
	Peak	Peak	Inflection point	Remote
**Beamline**	X12	X12	X12	X12
**Space group**	P6_1_22	C2	C2	C2
**Unit cell parameters**				
**a (Å)**	79.79	116.81	117.1	116.75
**b (Å)**	79.49	51.97	51.92	51.96
**c (Å)**	434.11	99.00	99.15	99.00
**β, γ (°)**	120.0	122.8	122.9	122.8
**Resolution range (Å)**	50.00–2.49	50.00–1.47	50.00–1.46	50.00–1.50
**Wavelength (Å)**	0.9799	0.9796	0.9894	0.9537
**Average redundancy**	15.0 (3.2)	2.9 (2.1)	4.4 (2.8)	3.1 (2.2)
**Unique reflections**	29302	82697	85761	77301
**Completeness (%)**	97.46 (75.5)	97.3 (81.6)	98.6 (87.3)	97.6 (84.2)
**Rmerge (%)**	7.7 (15.1)	4.9 (36.5)	5.2 (39.4)	4.5 (41.7)
**Average I/σ(I)**	29.0 (3.5)	18.9 (1.8)	21.0 (1.9)	16.8 (1.7)

Values in parentheses are for higher resolution shells (2.53–2.49 Å, 1.52–1.47 Å, 1.51–1.46 Å and 1.44–1.50 Å for Holo-pk, Apo-pk, Apo-ip and Apo-rm, respectively).

The structure solution unveils the presence of two independent polypeptide chains in the asymmetric unit of the ApoTmArgBP crystals. An analysis of the intermolecular contacts established by ApoTmArgBP molecules within the crystals, carried out by using the server PISA, clearly indicates that the these two independent molecules constitute a stable dimer. Indeed, the total buried area in the complex is 1387 Å^2^. This finding fully agrees with the characterization of the ApoTmArgBP oligomeric state in solution (see above). (residues 23–110 and 210–231) and lobe II (residues 116–203).

Similar to other arginine-binding proteins, ApoTmArgBP is folded into two distinct lobes, each consisting of a β-sheet core formed by five strands, surrounded by helices. β-sheets of lobes I (residues 23–110 and residues 210–231) and II (residues 116–203) present topologies β_2_β_1_β_3_β_5_β_4_ and β_8_β_7_β_9_β_6_β_10_, respectively. The two lobes are connected by two distinct segments (residues 111–115 and 204–209), a characteristics typical of class II SBPs [12]. In ApoTmArgBP, the two connecting segments form a short two-stranded β-sheet, which is stabilized by three main chain hydrogen bonds. In line with unliganded SBPs, the two lobes of the proteins are far away from each other and do not establish significant non-covalent interactions.

**Figure 4:**
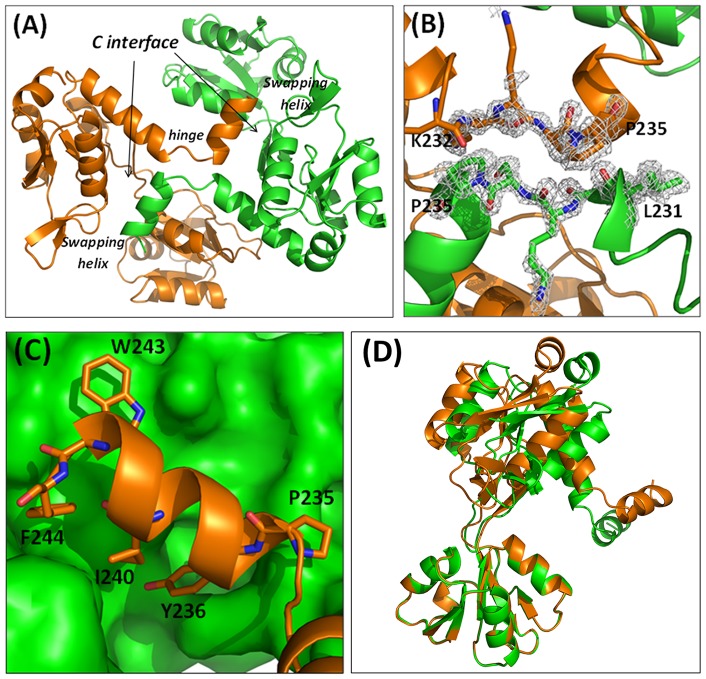
Domain-swapped dimer of ApoTmArgBP. (A) Cartoon representation of ApoTmArgBP swapping dimer. (B) Omit (Fo-Fc) map of the hinge region, contoured at 2σ. (C) Interactions mediated by the C-terminal helix. (D) Superposition of the chains A and B of ApoTmArgBP.

The analysis of the protein N- and C-termini unveils interesting and unexpected features. The very N-terminal residues of the construct used in the present study (residues 20–27) form a stable α-helix. This suggests that the transmembrane helical region (residues 4–21) extends in the periplasm maintaining its structure. The limited number of contacts of this helix with the rest of the protein suggests that this region is free to adopt alternative states upon the anchoring of the signal peptide to the membrane. The analysis of the C-terminus indicates that TmArgBP dimerization is caused by its 3D domain swapping (Figure 4A). Indeed, the two subunits in the dimer mutually exchange their C-terminal helix (residues 237–246). An important role in the swapping process is played by the hinge region 232-KKSPY-236 whose extended conformation hampers the association of the terminal helix with its own subunit and favors its anchoring the main body of the other polypeptide chain (Figure 4B). A comparison of the local sequence of the hinge region with those of other arginine binding proteins with known 3D structures (Figure 1) suggests that it is more conformationally restrained in TmArgBP. Indeed, the hinge region of TmArgBP presents a deletion that shortens the loop length, which is associated with the concomitant presence of a Pro residue. It is reasonable to assume that this specific motif is responsible for the domain swapping observed in TmArgBP.

According to the notation introduced by Eisenberg et al. [13] different contact surfaces may be identified in a 3D-swapping protein, as evidenced in Figure 4. The C-interface (closed interface) is the contact area formed by the swapping fragment with the main body of the other chain. This is expected to be present in the non-swapping monomer of the same or similar monomeric proteins. On the other hand, the O-interface (open interface) exclusively occurs in a 3D domain-swapped dimer and is not present in the closed monomeric form. In this framework, the C-interface of the ApoTmArgBP dimer reproduces the contacts between the C-terminal helix and the rest of the protein. This interface is stabilized by a strong salt bridge formed by the side chains of Asp56 of chain A with Lys242 of chain B (and *viceversa*). A weaker electrostatic interaction is formed by the side chains of Lys193 of one chain and Glu241 of the other chain. In addition, a number of hydrophobic interactions are detected at the C-interface. In particular, close contacts are established by the side chains of Tyr236, Trp243, Phe244, Leu239 and Ile240 from the C-terminal helix of one monomer with a concave cavity of the adjacent monomer, formed by Phe53, Leu57, and Phe112 (Figure 4C). On the other hand, only sporadic interactions are detected at the O-interface (Figure 4B). In contrast to the majority of assemblies characterized by domain swapping, the main bodies of the two subunits do not form any specific interaction. The only new interactions formed upon the formation of the swapped dimer are those involving the hinge peptide. A strong hydrogen bond between the two subunits is formed by the N main chain atom of Tyr236 of one chain with the oxygen atom of Leu231 of the other chain (and *viceversa*). In addition, the OH atom Tyr236 of the hinge region forms a hydrogen bond with the backbone oxygen atom of Tyr111. Therefore, the swapping dimer is essentially stabilized by interactions formed at the C-interface. Finally, it is worth noting that the two independent molecules of ApoTmArgBP display significant differences at the level of tertiary structure (Figure 4D). This indicates that this form is endowed with a significant flexibility (see also below).

### The crystal structure of HoloTmArgBP: molecular basis of arginine recognition by HoloTmArgBP

**Figure 5:**
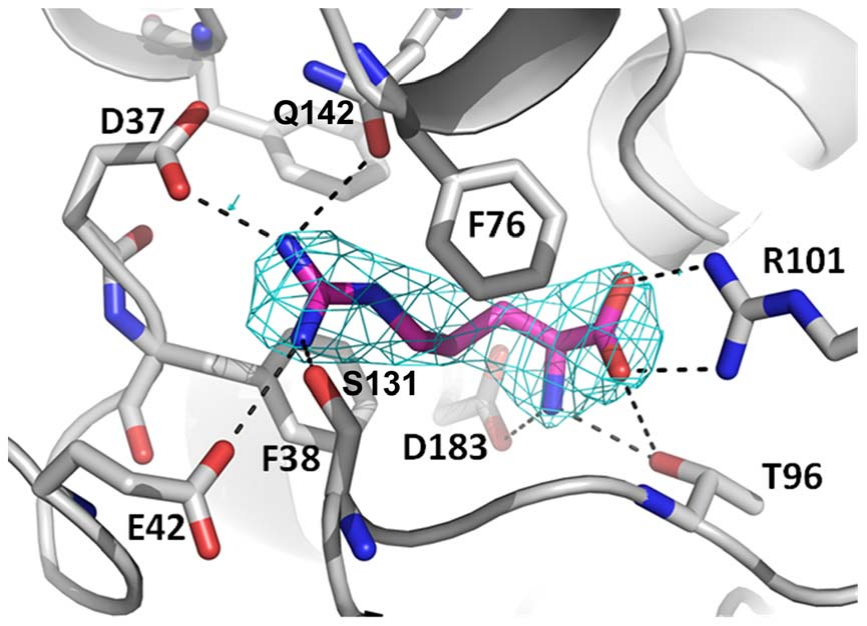
(2Fo-Fc) electron density map contoured around the arginine ligand (2.0 σ). Arginine interacting residues are highlighted.

In order to determine the structural basis of arginine recognition by TmArgBP we also determined the crystal structure of the bound form of the protein. We initially characterized the recombinant protein without adding any external amino-acid. For this form, a readily interpretable electron density throughout the entire structure was obtained by using the Single-wavelength Anomalous Dispersion method, (Table 2). Although crystallization was performed without adding exogenous arginine, the inspection of maps corresponding to the putative arginine binding pocket clearly unveiled the presence of an elongated electron density corresponding to an arginine (Figure 5). This is indicative of the tight affinity of TmArgBP for arginine at room temperature.

**Table 2:** Refinement statistics.

	HoloTmArgBP	ApoTmArgBP
**Resolution range (Å)**	15.00–2.49	15.00–1.47
**Asymmetric unit R (%)**	Dimer 17.8	Dimer 14.9
**R_free_ (%)**	22.8	20.0
**No. of residues**	452	454
**No. of ligand molecole**	2	0
**No. of water molecules**	470	820
**Mean B value (Å^2^)**	30.5	21.1
**R.m.s. deviations**		
**Bond lengths (Å)**	0.018	0.017
**Bond angles (°)**	1.9	1.8

Values in parentheses are for higher resolution shells (2.53–2.49 Å and 1.52–1.47 Å for Holo and ApoTmArgBP, respectively).

**Figure 6:**
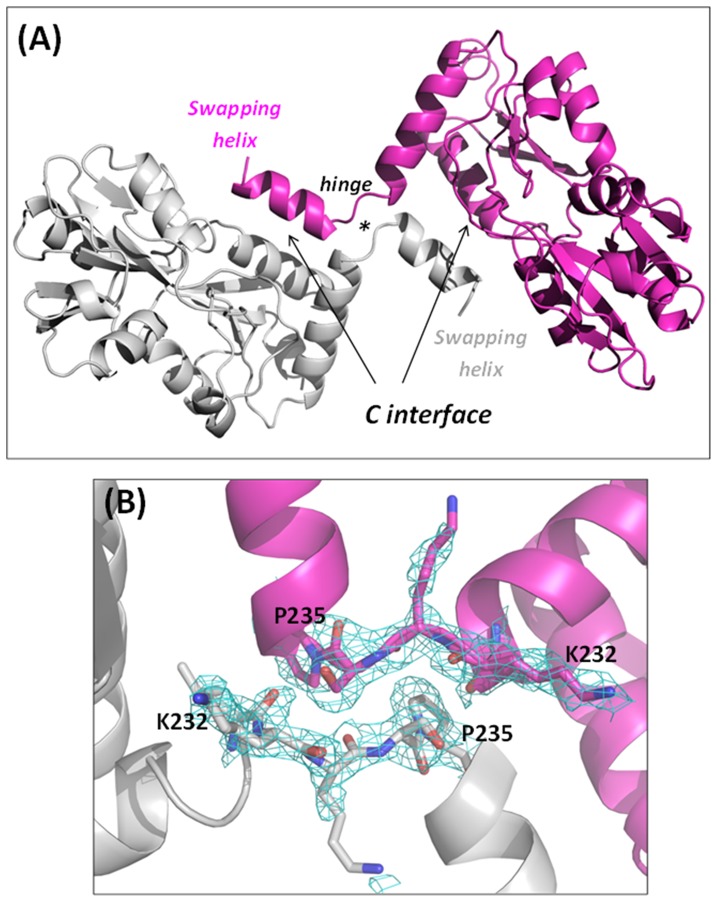
Swapping dimer of HoloTmArgBP. (A) Cartoon representation of HoloTmArgBP domain-swapped dimer. (B) Omit (Fo-Fc) map of the hinge region contoured at 2σ.

The crystals of HoloTmArgBP also contain two molecules in the asymmetric unit. The two molecules are virtually identical, with a root mean square deviation (RMSD) value, calculated on backbone atoms, of 0.9 Å. Similar to the apo form, these two molecules form a tight dimer, which buries an area of 978 Å^2^. The inspection of the electron density maps clearly shows that dimer formation occurs through swapping of the C-terminal helix, with the hinge region located between Lys232 and Tyr236 (Figure 6). The C- and the O-interfaces of the holo form are, in terms of H-bonds and hydrophobic interactions, virtually identical to those detected for ApoTmArgBP. As a result, the formation of the interface causes, according to PISA [14], a strong gain of free energy of solvation (ΔG = −21.1 kcal/mol).

**Figure 7:**
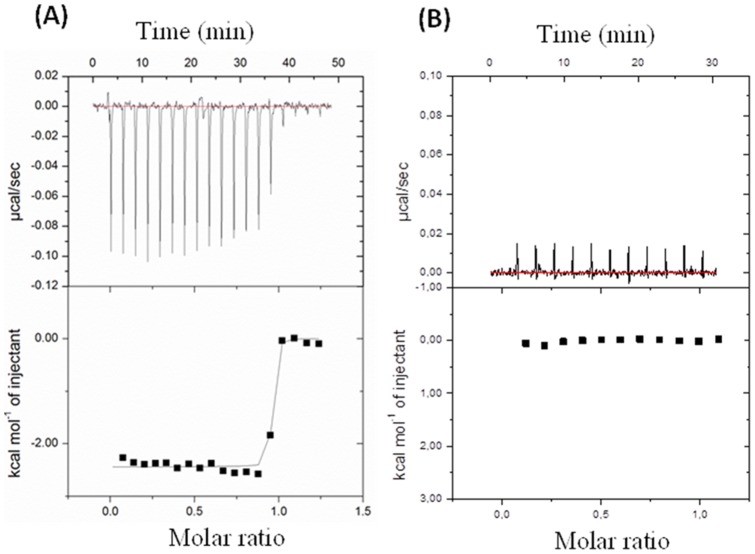
Isothermal titration calorimetry experiments with (A) arginine and (B) glutamine. Top panels report raw data for the titrations at 25°C, whereas bottom panels report integrated heats of binding obtained from the raw data after subtracting the heats of dilution. The solid line (in A) represents the best curve fit to the experimental data using the ‘one set of sites’ model from MicroCal Origin.

The inspection of the TmArgBP binding pocket shows that the protein anchors arginine through a variegate ensemble of interactions that include hydrogen bonds, salt bridges and hydrophobic interactions. These interactions cage the arginine ligand by tightly anchoring both its backbone and the side chain, thus making it fully solvent inaccessible. Backbone interactions involve a bifurcated salt bridge between the carboxyl end of arginine and the side chain of Arg101 and a salt bridge between the backbone nitrogen and the side chain of Asp183 (Figure 5). The aliphatic portion of arginine side chain is sandwiched between the two aromatic rings of Phe38 and Phe76, whereas the guanidine group is involved in salt bridges with Asp37 and Glu42, as well as hydrogen bonds with the side chains of Gln142 and with Ser131 (Figure 5). The binding of arginine ligand to ApoTmArgBP was quantified using Isothermal Titration Calorimetry (ITC) (Figure 7). Binding isotherms for the interaction of ApoTmArgBP with arginine, measured at pH 8.0, were characterized by exothermic heats of binding which decreased in magnitude with successive injections until saturation was achieved (Figure 7A). Consistent with the several interactions observed in the crystal structure of TmArgBP complex, our data indicate a strong enzyme-inhibitor binding, with K_D_ in the low nanomolar range (1.3±0.9 nM). On the other hand, we observed that ApoTmArgBP is unable to bind glutamine (Figure 7B). This result suggests that the salt-bridge interactions with Asp56 and Glu61 established by the guanidine group of Arg are essential for amino acid binding.

### ApoTmArgBP vs HoloTmArgBP: tertiary and quaternary structure variations

The comparison of the two independent molecules present in the structures of ApoTmArgBP and HoloTmArgBP indirectly indicates that the two forms are endowed with distinct flexibility at tertiary structure level. The two molecules of the unliganded forms display significant differences whereas the holo molecules are virtually unchanged. Using the program DynDom [15], we computed a difference in the closure angle between lobe I and lobe II of 20° for the apo form (Figure 4D), whereas no difference was detected for the holo form. These results show that the apo form is endowed with a larger intrinsic flexibility.

**Figure 8:**
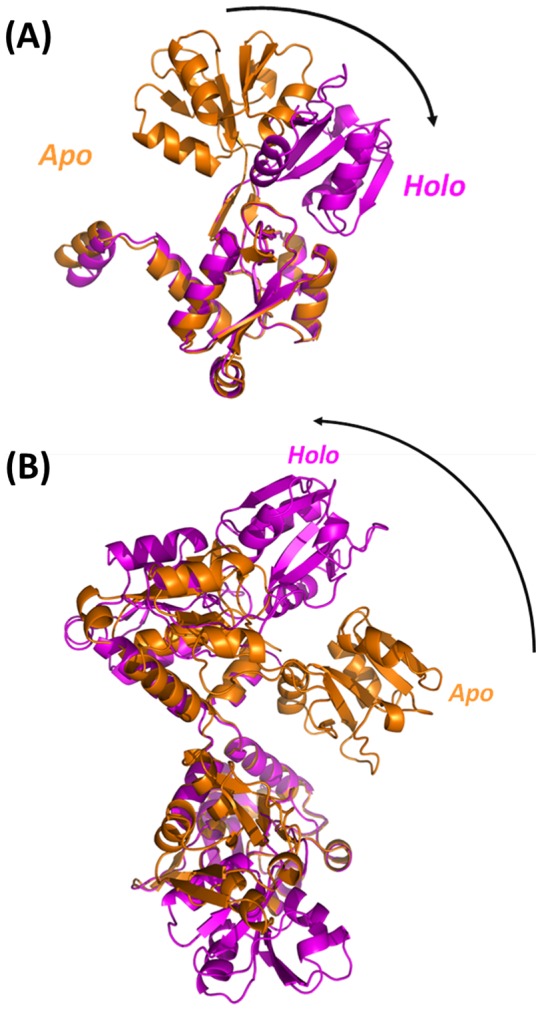
Variation of tertiary (A) and quaternary (B) structures of ApoTmArgBP (orange) and HoloTmArgBP (magenta). In both panels, overlapped regions are 22–104 and 206–243 of lobe I. The arrows highlight the conformational changes occurring upon arginine binding.

Although the basic secondary structure elements are preserved in the apo and holo forms of the protein, huge variations are observed at tertiary structure level. Indeed, RMSD values, calculated on the Cα atoms of each monomer are as high as 8.4 Å. The comparison of the tertiary structure of the liganded and unliganded structure demonstrates that the binding of the arginine ligand brings the two lobes together. The remarkable domain closure in HoloTmArgBP requires rotations of lobe II towards lobe I of 82°–84° starting from the two monomers of ApoTmArgBP (Figure 8A). Consistently, when single lobes of the two forms are overlapped, RMSD values drop to 0.5 Å for both lobes.

The observed variations of the tertiary structures of ArgBP upon arginine binding have a strong effect on the protein quaternary structure (Figure 8B). Interestingly, ApoTmArgBP, which is characterised by a less compact and more flexible tertiary structure, presents a more compact quaternary structure. Indeed, the C-terminal helix of Apo-TmArgBP forms interactions with residues at the closed interface between the two lobes of the adjacent monomer (Figure 4), an interface which is not accessible in the Holo form, since locked by the arginine ligand. Consistently, the formation of the apo dimer covers a larger interface area than observed for HoloTmArgBP (1383 *versus* 978 Å^2^), albeit with a similar gain of the free energy of solvation.

## Discussion

The determination of the three-dimensional structure of the apo and holo forms of TmArgBP reveals, along with predictable features, some unexpected findings. The secondary structural elements and protein organization in distinct subdomains (lobes) connected by two joining fragments confirm that TmArgBP is a type II SBP. In addition, the length of these joining regions classifies TmArgBP among cluster F according to the definition proposed by Poolman and co-workers [12]. The tertiary structure closure associated with the arginine binding is well described by the Venus Fly-trap mechanism observed for other SBPs [5], [12]. The comparison of the apo and the holo forms of TmArgBP shows that the structural re-organization upon substrate binding is associated with a domain movement as large as 80°.

One novel element emerged from the present study is that the protein forms a dimer through the swapping of the C-terminal helix. This dimer is characterized by rather loose quaternary structure organization (Figures 4 and 6). Indeed, the two subunits are essentially held together by interactions formed by the swapping C-terminal helices. In contrast, the main bodies (residues 20–233) of the two chains do not establish significant interactions. This feature suggests that both the apo and the holo forms are endowed with a remarkable flexibility at the quaternary level. Molecular dynamics simulations carried out on the swapping dimers of RNase A have clearly indicated that interactions formed by the main bodies of the proteins play a crucial role in dictating the overall flexibility of these assemblies [16]. Indeed, the RNase A C-terminal swapping dimer, which presents a loose interface [17], is highly flexible [16] whereas the N-terminal swapping dimer presents a tight association of the two subunits and is rather rigid [18], [19]. In this scenario, it is likely that the dynamic behavior of both ApoTmArgBP and HoloTmArgBP resembles the one observed for RNase A C-terminal swapping dimer. It is important to note that solution studies presented here clearly show that the peculiar stability of TmArgBP is not confined to the individual subunits of the protein but also extends to the oligomeric association. Therefore, domain swapping confers TmArgBP a combination of plasticity and stability that is not achievable in canonical (non-swapped) dimeric associations. Moreover, the preservation of the swapping in both the apo and the holo forms makes TmArgBP structure an unusual example of the preservation of domain swapping despite giant variations of the tertiary structure. It is interesting to note that TakP, a unrelated SBP from a TRAP transporter, also dimerizes through the swapping of the C-terminal helix [20]. In this case, however, tertiary structure variations associated with ligand binding are very limited. The oligomerization through domain swapping exhibited by TmArgBP also provides a rationale for the observed ability of the protein to form higher aggregates [7], [9]. These states may be achieved through a mutual swapping of the C-terminal helix that is not limited to two subunits but involves three (trimers) or four (tetramers) protein molecules, as found in RNase A [13], [21], [22].

The observation that TmArgBP dimerizes through the exchange of the C-terminal helix leads us to question the sequence determinants of swapping. Literature studies have shown that, among other factors, the presence of Pro residues and/or the occurrence of deletions in the hinge regions favor the swapping *via* a destabilization of the monomeric form [23], [24]. The analysis of TmArgBP hinge sequence shows the occurrence of a deletion that is concomitant with the presence of a Pro residue (Pro236). This feature is not found in other ArgBPs with a known 3D structure (Figure 1). An analysis of protein sequences indicates that TmArgBP shares a similar hinge sequence only with very close homologs. Indeed, the most distant protein (NCBI Reference Sequence YP_001305722) that presents the same deletion and the Pro residue in the hinge peptide show a sequence identity with TmArgBP of 60%. In evolutionary terms, this may suggest that the propensity of this protein to dimerize through domain swapping is a recently acquired property.

**Figure 9:**
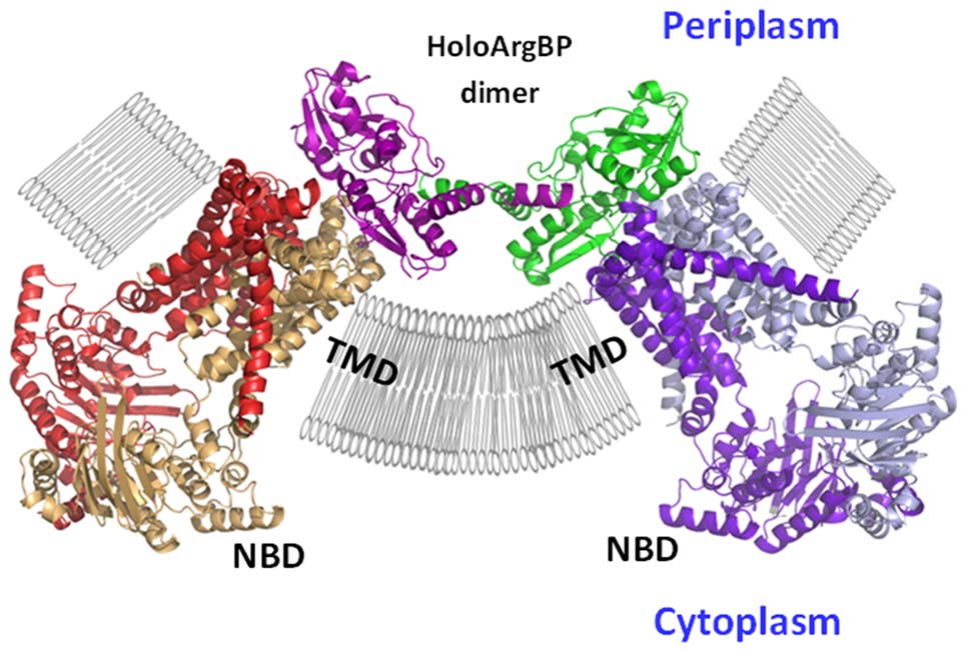
A model for ABC cassette bound to HoloTmArgBP swapping dimer.

It is well know that the vast majority of SBPs operate as monomers as only sporadic examples of dimeric SBPs [12], essentially related to TRAP receptors, have been reported [20], [25], . In this scenario, the observation that TmArgBP is able to form a stable dimer leads to question about the relevance of the oligomeric state of TmArgBP on its biological function. We checked whether the two subunits of the TmArgBP dimer could simultaneously bind the ABC cassette system. The interactions of TmArgBP with an ABC transporter was modeled by following the procedure adopted by Vahedi-Faridi et al. [28] for ArtJ and using the complex between molybdate/tungstate ABC transporter and its cognate SBP [29] as a model template. The overall shape of this speculative complex (Figure 9) is compatible with the simultaneous binding of TmArgBP swapping dimer with two independent ABC transporter modules. The analysis of this complex also provides some preliminary indications on regions of TmArgBP involved in the transporter recognition. Based on analogy with ArtJ, there are two main regions comprised of residues 42–51 in lobe I and residues 171–177 in lobe II that play a major role in this process. Of particular interest is the observation that Glu163 of ArtJ, which was found by mutagenesis analyses to be a key player in this recognition process [28], is conserved in TmArgBP (Glu171).

The availability of the 3D model of TmArgBP also offers the possibility to relate the extraordinary thermostability of the protein to specific features of its structure. We compared sequence and structural features of TmArgBP with arginine binding proteins isolated from mesophilic organisms with known 3D structures. In particular, we selected the arginine binding proteins AbpA isolated from *Streptococcus pneumoniae* (PDB code 4I62) and STM4351 isolated from *Salmonella enterica* (PDB code 2Y7I) [30]. It is commonly accepted that electrostatic interactions frequently play a major role in protein structure stabilization [31]. In this framework, we initially considered the overall content of polar versus charged amino acids, since it has been shown that in thermostable proteins there is an accumulation of charged residues associated with a reduction of polar residues [32], [33]. Our analysis indicate that TmArgBP contains a higher percentage (30.8% vs 25.2%) of charged residues (Lys, Arg, Glu, and Asp) and a lower amount (14.5% vs 21.0%) of polar residues (Asn, Ser, Gln, and Thr) when compared to STM4351. This trend is less clear in the comparison with AbpA, as TmArgBP maintains a lower amount of polar residues (14.5% vs 21.8%), but with similar percentages of charged residues (30.8% vs 29.5%). This analysis agrees with studies carried out on other hypertermophilic enzymes, that also exhibit a remarkable thermostability [32], [33]. We next evaluated the occurrence and the frequency of specific interactions between charged residues that are commonly shown to play a role in the thermal stabilization of protein structure. In particular, our analysis found the occurrence of a larger number of salt bridges in TmArgBP (11 bridges) compared to STM4351 (5 bridges) and AbpA (8 bridges). These observations indicate that electrostatic interactions likely play a significant role in the stabilization of the protein.

This study also elucidates the molecular recognition mechanism of TmArgBP for arginine at atomic level. The protein binds the substrate through an intricate network of interactions, which results in a high affinity of binding at room temperature (K_D_ in the nanomolar range). Although this may appear in contrast with previous reports [7], the analysis of the 3D structure reconciles all data. Indeed, SPR experiments indicated a much lower affinity (K_D_ of 17 µM) of the protein for a rather complex arginine-containing peptide [9]. The lower affinity for this peptide can be easily explained by considering that the protein strongly grasps both charged amino and carboxyl ends of the aminoacid. Very recently, it has been reported that mutations of residues of the TmArgBP binding pocket, that were specifically designed to achieve a fluorescence variation upon substrate binding, lead to variants that bind arginine with micromolar affinities [7]. The analysis of TmArgBP binding pocket suggests that the replacements of Gly94, Met95, Gln116, and Thr146 with a bulkier Trp strongly reduces the affinity for the substrate through a partial occupation of the binding site or by inducing a local destabilization of the structure (Figure S6 in File S1).

Taken together our data represent a significant advancement for the design and the characterization of TmArgBP mutants that may be used for constructing arginine sensors. It is worth mentioning that the use of the wild-type protein is not suited for this purpose since no variation of Trp florescence is observed upon arginine binding. Consistently, the local environment of Trp243 does not change in the structures of the apo and the holo forms here described, despite the huge structural rearrangements of the protein structure upon arginine binding. In both cases, this residue is buried by the interactions established by the swapped C-terminal region with the main body of the protein. The availability of an accurate 3D model is a powerful tool for the design of new TmArgBP variants, by fine-tuning substrate affinity and fluorescence signal, that are better suited for biotechnological applications.

## Materials and Methods

### Protein sample preparation

The wild-type protein used in these studies includes the residues 20–246 of the protein sequence (UniProt code Q9WZ62). This region corresponds to the entire protein deprived of the signal sequence for its perisplasmic exporting. TmArgBP was expressed by using *E. coli* Rosetta(DE3)2 cells following the procedure described in Luchansky et al. [9] and Ruggiero et al. [34]. Since the expressed protein is obtained in the arginine bound state (HoloTmArgBP), its ligand-free form (ApoTmArgBP) was prepared using protocols previously reported [9], [34].

### Light scattering experiments

Purified proteins (HoloTmArgBP and ApoTmArgBP) were analyzed by size-exclusion chromatography connected to a triple-angle light scattering detector equipped with a QELS module (quasi-elastic light scattering). Specifically, protein samples of 500 µg were loaded on a S200 10/30 column, equilibrated in 20 mM Tris–HCl (pH 8.0) and 150 mM NaCl. A constant flow rate of 0.5 ml/min was applied. Elution profiles were detected by a Shodex interferometric refractometer and analyzed using a miniDawn TREOS light scattering system (Wyatt Instrument Technology Corp.). Data were processed using the Astra 5.3.4.14 software package.

### Denaturing and native gel electrophoresis analysis

The stability of the TmArgBP dimer was assessed by SDS polyacrylamide gel electrophoresis (SDS-PAGE). The samples were prepared in 10% (v/v) SDS and denatured at 373K for 5 minutes.

The oligomerization state of protein samples was also analyzed using native polyacrylamide gel electrophoresis (N-PAGE). In this case, purified proteins were diluted in native-sample buffer (0.06 M TrisHCl, 2.5% (v/v) glycerol, 0.001% (w/v) Bromophenol Blue, pH 6.8) and loaded on 10% (v/v) N-PAGE. The electrophoresis was carried out for 2 hours at 25 mA.

### Isothermal titration calorimetry

The interaction of ApoTmArgBP with arginine or glutamine was investigated at 298 K by isothermal titration calorimetry using a MicroCal ITC200 calorimeter (GEHelthcare, Milan) calibrated with standard electrical pulses. All solutions were degassed by stirring under vacuum before use. In these experiments, 18 consecutive injections of 2 µL aliquots of a 1.25 mM arginine solution were added to the calorimeter cell (0.280 mL) containing 0.05 mM of apoTmArgBP_20–246_ at intervals of 150 seconds. To minimize the contribution of heat of dilution to the measured heat change, protein and ligand solutions were prepared in the same buffer (50 mM TrisHCl, 150 mM NaCl, pH 8). In order to ensure proper mixing after each injection, a constant stirring speed of 1000 rpm was maintained during the experiment. Data were analyzed using a ‘one set of sites’ binding model.

### Protein expression, purification and crystallization

The failure to solve the crystal structure of TmArgBP by molecular replacement [34], prompted us to prepare a selenomethionine derivative (Se-Met) of the protein. The Se-Met derivative of TmArgBP was expressed by using *E. coli* Rosetta(DE3)2 cells in 1L of minimal media (M9) enriched with the following components: 0.4% (w/v) glucose, 1 mM MgSO_4_, 0.1 mM CaCl_2_, 50 ugL^−1^ ampicillin, 33 ug L^−1^ chloramphenicol, 100 ugL^−1^ thiamine at 37°C. After reaching an OD_600_ of 0.7, an aminoacid mix (50 mg L^−1^ Ile, Leu and Val and 100 mg L^−1^ of Phe, Thr, and Lys) was added to the bacterial culture. After equilibration, 60 mg L^−1^ of seleno-L-methionine were added and the induction was performed by adding 0.5 mM IPTG. The labelled protein was purified as previously described [34]. The homogeneity of the protein was evaluated by SDS–PAGE analysis. The molecular mass of the purified protein was checked by mass spectrometry and no proteolysis of the protein was detected. The ligand-free form of TmArgBP was prepared using procedures previously reported [34].

Crystallization of both ligand-bound and ligand-free SeMet TmArgBP was performed at 293 K by hanging-drop vapour-diffusion methods. In previous studies, HoloTmArgBP was crystallized in the presence of PEG 10,000 as a precipitant, whereas ApoTmArgBP was crystallized by using PEG 3,350 [34]. However, a further screening/optimization of crystallisation conditions was achieved for the Se-Met derivatives of both HoloTmArgBP and ApoTmArgBP, using the PEG/Ion screen (Hampton Research) and then the Additive screen (Additive Formulation, Hampton Research). New crystallization conditions were found for both forms. The best crystals of HoloTmArgBP were obtained using a protein concentration of 25 mg mL^−1^ and 0.2 M Potassium acetate, 20% (w/v) Polyethylene glycol 3,350 using LDAO as an additive [34]. These crystals diffracted to 2.49 Å at the X12 beamline, DESY, Hamburg (Table 1). Crystals of the ligand-free TmArgBP were obtained with the same procedure adopted for the native protein. Best crystals grew in 16–20 mg mL^−1^ protein solution and in 25% w/v PEG 3,350 and 0.1 M Sodium Acetate trihydrate (pH 4.6). These crystals diffracted to 1.5 Å and belonged to the C2 space group (Table 1).

### Data collection, processing and structure determination

Diffraction data for the Se-Met derivatives of holo and apo forms of TmArgBP were collected at the X12 synchrotron beamline, DORIS storage ring, DESY (Hamburg, Germany) at 100 K. Cryoprotection of the crystals was achieved by a fast soaking in a solution containing ethylene glycol to a final concentration of 14% (v/v). Because of a large unit cell, the diffraction data of HoloTmArgBP were collected using a small rotation angle of 0.1°. To avoid the overlap of diffracted intensities for HoloTmArgBP the resolution of the collected dataset was limited at 2.49 Å, although spots were detectable at resolution as high as 2.0 Å. The data sets of both forms were scaled and merged using the HKL2000 program package [35].

Multi-wavelength anomalous diffraction (MAD) experiments were carried on Se-Met labelled crystals. For peak and inflection wavelength determination, fluorescence scans were recorded for both holo and apo TmArgBP crystals. For ApoTmArgBP, data sets were collected at three wavelengths (peak, inflection and remote), optimised for Se-Met. For HoloTmArgBP, diffraction data were collected only at the wavelength corresponding to the peak. Statistics of data collection are reported in Table 1.

### Structure determination and refinement

The Auto-Rickshaw pipeline was adopted to determine both the structures of holo and apo forms [36], using the SAD (Single-wavelength Anomalous Dispersion) and MAD methods, respectively. The initial set of phases was improved by using solvent-flattening and phase extension methods. Manual modelling was performed using Coot [37].

Crystallographic refinement of both structures was carried out against 95% of the measured data using the ccp4i program suite. The remaining 5% of the observed data, which was randomly selected, was used in R_free_ calculations to monitor the progress of refinement. Non crystallographic restraints were applied in REFMAC [38] with medium restraints for main-chain atoms and loose restraints for side-chain atoms. Water molecules were incorporated into the structure in several rounds of successive refinement. For the apo form the entire construct sequence (residues 20–246) was modelled in the final electron density. Due to the lower resolution of data, the two C-terminal residues could be not modelled for the holo form. The basic stereochemistry of the model was checked by using the program PROCHECK. We evaluated the occurrence of some correlations between geometrical parameters that are typically detected in highly accurate protein structures and are not biased by restraints in the crystallographic refinement. In particular, we checked the dependence on the ψ angle (i) of the NC^α^C bond angle [39], [40], (ii) of peptide bond planarity [41] and (iii) of the carbon carbonyl pyramidalization ϑc [39], [40], [42] (Figures S1–S4 in File S1). For the high resolution apo structure, the NC^α^C/ψ and the peptide planarity versus ψ correlations are clearly detected. Surprisingly, the dependence of the peptide planarity on the ψ angle was also detected in the structure of the holo form despite the relatively low resolution of the dataset. This observation may be explained by considering that the crystals of the holo form diffracted at a resolution beyond the resolution limit of the dataset and that the large unit cell prevented the collection at higher resolution (see above). The structures of the HoloTmArgBP and ApoTmArgBP have been deposited in the PDB with the codes 4PSH and 4PRS, respectively.

### Notation

Throughout the text, the residue numbering refers to the full-length protein 1–246.

## Supporting Information

File S1 Contains the following files: **Figure S1.** Distribution of NCαC angles in residues located in β-sheets (A) and α-helices (B) of ApoTmArgBP. As found in well-refined high resolution structures the value of the angle is, on average, larger in α-helical residues. Indeed, the average value of the NCαC angle for residues located in α-helices and β-sheets is 111.2 and 108.9°, respectively. A similar trend is observed for HoloTmArgBP, although differences are less pronounced. In this case, the average value of the NCαC angle for residues located in α-helices and β-sheets is 111.4 and 110.1°, respectively. **Figure S2.** Dependence of the peptide planarity, expressed as Δω =  ω-180°, on the ψ dihedral angle for ApoTmArgBP. As shown in panel A some variations of the peptide bond planarity are observed. The analysis of Δω in the region 75° < ψ <105° confirms the average positive value for this parameter detected in atomic resolution protein structures (B). The number of points is, however, rather low. The average value of Δω in the region 135° < ψ <165° is positive, in line with what found in atomic resolution protein structures (C). **Figure S3.** Dependence of the peptide planarity, expressed as Δω =  ω-180°, on the ψ dihedral angle for HoloTmArgBP. Despite the lower resolution of the HoloTmArgBP compared to ApoTmArgBP the two proteins exhibits similar trends (Figure S2). **Figure S4.** Dependence of the carbon carbon carbonyl pyramidalization ϑc on the ψ dihedral angle for ApoTmArgBP. Although some variations of the pyramidalization are observed the trends are not very significant (A). The analysis of ϑc in the region 75° < ψ <105° confirms an average positive value for ϑc detected in atomic resolution protein structures (A). The number of points is, however, too low. The average value of ϑc in the region 135° < ψ <165° is positive, in line with what found in atomic resolution protein structures (C). However, the average ϑc value (0.9°) is too low to be considered significant. **Figure S5.** Prediction of the transmebrane regions of TmArgBP obtained by using the server TMHMM (http://www.cbs.dtu.dk/services/TMHMM/). For the sake of clarity, only the results related to the first 120 residues of the sequence are shown. **Figure S6.** Modeling of TmArgBP mutants that were mutated designed to achieve a fluorescence variation upon substrate binding (see the main text for details). The modeling was performed by using the structure of HoloTmArgBP as template. The replacement of Gly94 and Met95, with a bulkier Trp side chain, directly affects the binding site. On the other hand, the replacement of Gln116 and Thr146 likely induces a local destabilization of the protein structure.
(PDF)
