# Supplementary material for: A Loose Domain Swapping Organization Confers a Remarkable Stability to the Dimeric Structure of the Arginine Binding Protein from Thermotoga maritima
Source: PLoS One. 2014 May 15;9(5):e96560. doi: 10.1371/journal.pone.0096560 (PMC4022495; doi:10.1371/journal.pone.0096560)
Supplement: File S1 — Contains the following files: Figure S1. Distribution of NCαC angles in residues located in β-sheets (A) and α-helices (B) of ApoTmArgBP. As found in well-refined high resolution structures the value of the angle is, on average, larger in α-helical residues. Indeed, the average value of the NCαC angle for residues located in α-helices and β-sheets is 111.2 and 108.9°, respectively. A similar trend is observed for HoloTmArgBP, although differences are less pronounced. In this case, the average value of the NCαC angle for residues located in α-helices and β-sheets is 111.4 and 110.1°, respectively. Figure S2. Dependence of the peptide planarity, expressed as Δω = ω-180°, on the ψ dihedral angle for ApoTmArgBP. As shown in panel A some variations of the peptide bond planarity are observed. The analysis of Δω in the region 75° < ψ <105° confirms the average positive value for this parameter detected in atomic resolution protein structures (B). The number of points is, however, rather low. The average value of Δω in the region 135° < ψ <165° is positive, in line with what found in atomic resolution protein structures (C). Figure S3. Dependence of the peptide planarity, expressed as Δω = ω-180°, on the ψ dihedral angle for HoloTmArgBP. Despite the lower resolution of the HoloTmArgBP compared to ApoTmArgBP the two proteins exhibits similar trends (Figure S2). Figure S4. Dependence of the carbon carbon carbonyl pyramidalization ϑc on the ψ dihedral angle for ApoTmArgBP. Although some variations of the pyramidalization are observed the trends are not very significant (A). The analysis of ϑc in the region 75° < ψ <105° confirms an average positive value for ϑc detected in atomic resolution protein structures (A). The number of points is, however, too low. The average value of ϑc in the region 135° < ψ <165° is positive, in line with what found in atomic resolution protein structures (C). However, the average ϑc value (0.9°) is too low to be considered significant. Figure S5. [file pone.0096560.s001.pdf]

Supporting Information File S1

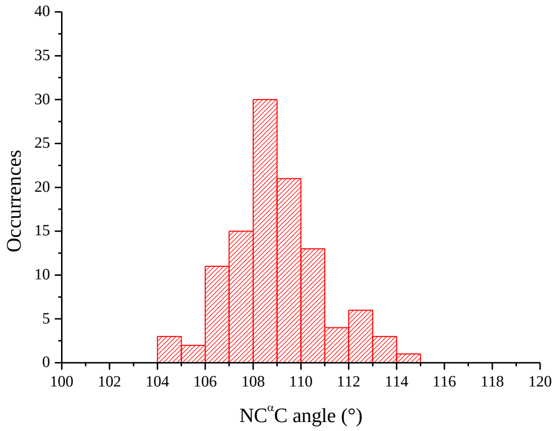

A

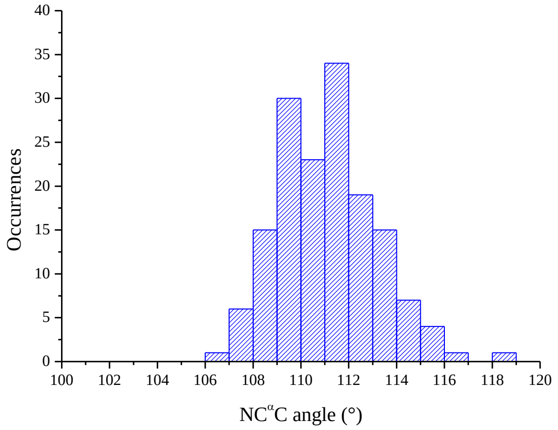

B

Figure S1.

Distribution of NC$^{\alpha}$C angles in residues located in β-sheets (A) and α-helices (B) of ApoTmArgBP. As found in well-refined high resolution structures the value of the angle is, on average, larger in α-helical residues. Indeed, the average value of the NC$^{\alpha}$C angle for residues located in α-helices and β-sheets is 111.2 and 108.9°, respectively. A similar trend is observed for HoloTmArgBP, although differences are less pronounced. In this case, the average value of the NC$^{\alpha}$C angle for residues located in α-helices and β-sheets is 111.4 and 110.1°, respectively.

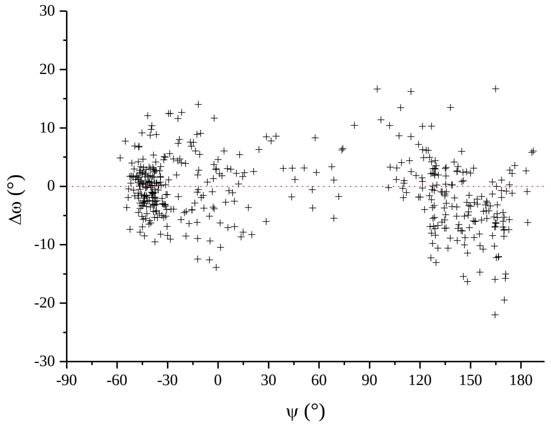

A

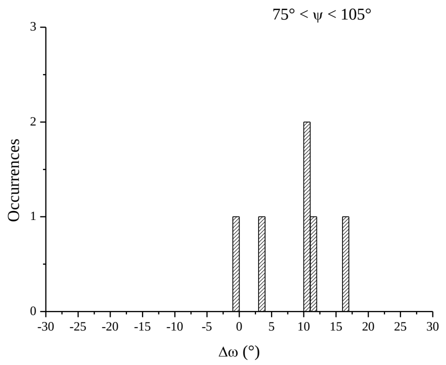

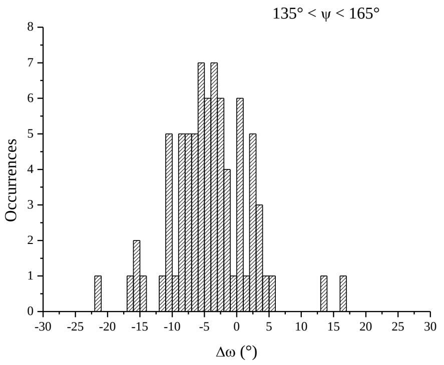

B

C

Figure S2.

Dependence of the peptide planarity, expressed as Δω= ω-180°, on the ψ dihedral angle for ApoTmArgBP. As shown in panel A some variations of the peptide bond planarity are observed. The analysis of Δω in the region 75° < ψ < 105° confirms the average positive value for this parameter detected in atomic resolution protein structures (B). The number of points is, however, rather low. The average value of Δω in the region 135° < ψ < 165° is positive, in line with what found in atomic resolution protein structures (C).

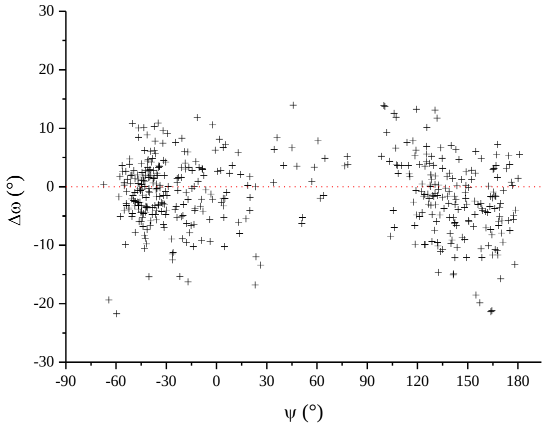

A

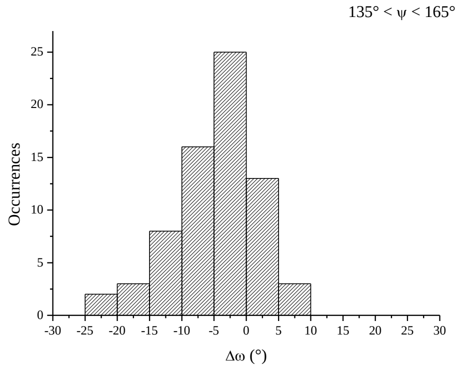

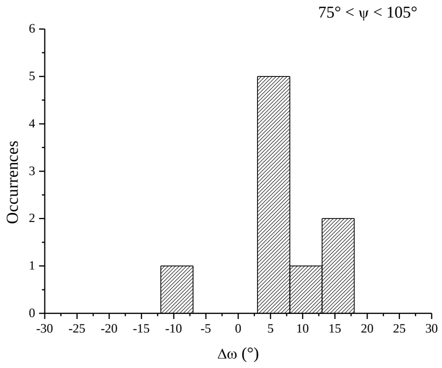

B

C

Figure S3.

Dependence of the peptide planarity, expressed as $\Delta\omega = \omega\text{-}180°$, on the ψ dihedral angle for HoloTmArgBP. Despite the lower resolution of the HoloTmArgBP compared to ApoTmArgBP the two proteins exhibits similar trends (Figure S2)

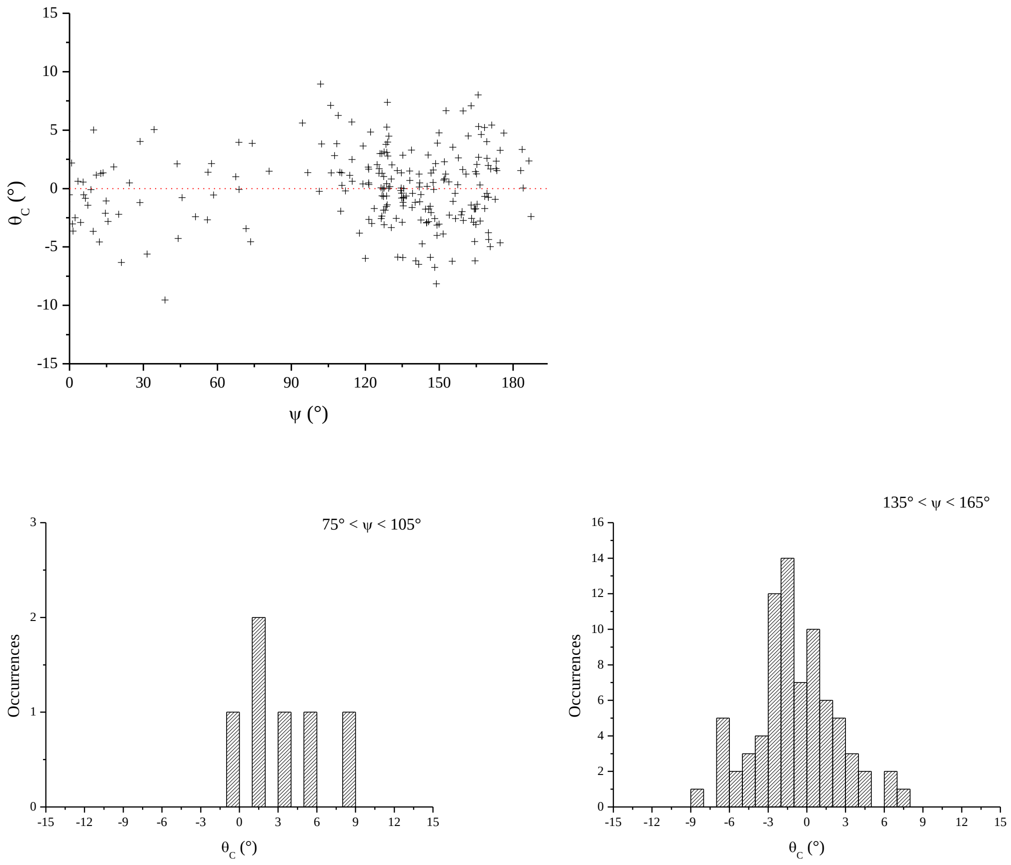

Figure S4.

Dependence of the carbon carbon carbonyl pyramidalization ϑc on the ψ dihedral angle for ApoTmArgBP. Although some variations of the pyramidalization are observed the trends are not very significant (A). The analysis of ϑc in the region $75° < \psi < 105°$ confirms an average positive value for ϑc detected in atomic resolution protein structures (A). The number of points is, however, too low. The average value of ϑc in the region $135° < \psi < 165°$ is positive, in line with what found in atomic resolution protein structures (C). However, the average ϑc value (0.9°) is too low to be considered significant.

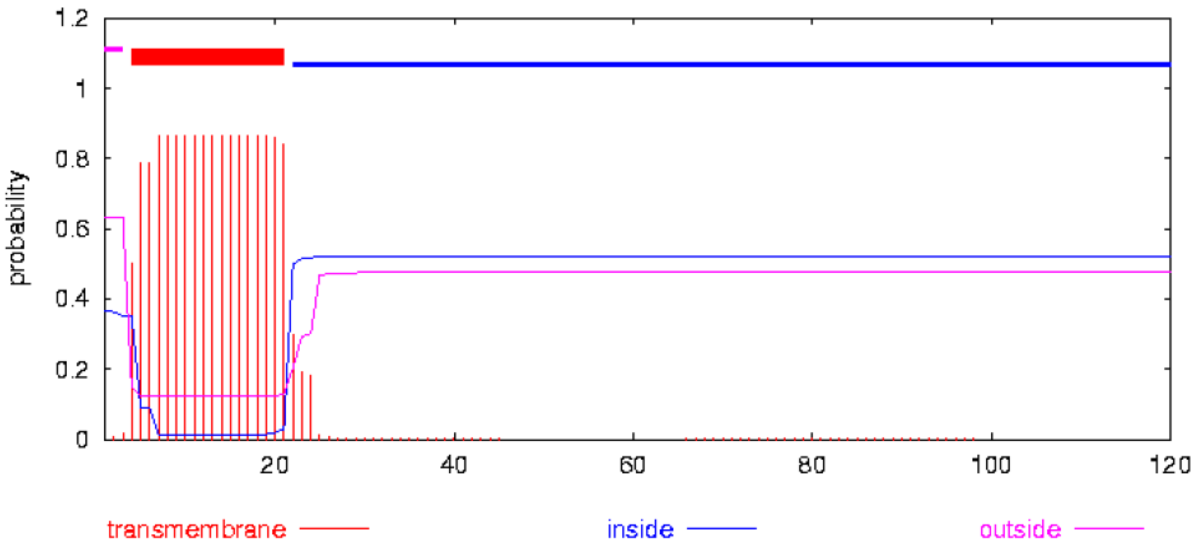

Figure S5.
Prediction of the transmebrane regions of TmArgBP obtained by using the server TMHMM (http://www.cbs.dtu.dk/services/TMHMM/). For the sake of clarity, only the results related to the first 120 residues of the sequence are shown.

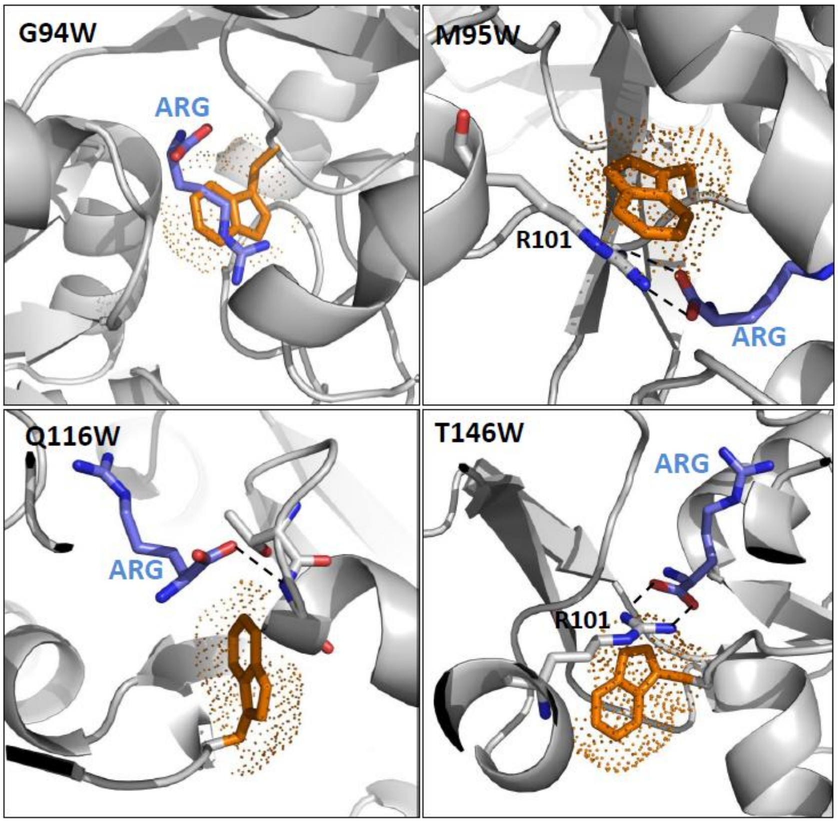

Figure S6.
Modeling of TmArgBP mutants that were mutated designed to achieve a fluorescence variation upon substrate binding (see the main text for details). The modeling was performed by using the structure of HoloTmArgBP as template. The replacement of Gly94 and Met95, with a bulkier Trp side chain, directly affects the binding site. On the other hand, the replacement of Gln116 and Thr146 likely induces a local destabilization of the protein structure.
